# A CARP-1 functional mimetic loaded vitamin E-TPGS micellar nano-formulation for inhibition of renal cell carcinoma

**DOI:** 10.18632/oncotarget.20650

**Published:** 2017-09-05

**Authors:** Vino T. Cheriyan, Hashem O. Alsaab, Sreeja Sekhar, Caitlin Stieber, Prashant Kesharwani, Samaresh Sau, Magesh Muthu, Lisa A. Polin, Edi Levi, Arun K. Iyer, Arun K. Rishi

**Affiliations:** ^1^ John D. Dingell VA Medical Center, Detroit, Michigan, 48201, USA; ^2^ Department of Oncology, Barbara Ann Karmanos Cancer Institute, Wayne State University School of Medicine, Detroit, Michigan, 48201, USA; ^3^ Department of Pathology, Wayne State University School of Medicine, Detroit, Michigan, 48201, USA; ^4^ Molecular Therapeutics Program, Barbara Ann Karmanos Cancer Institute, Wayne State University School of Medicine, Detroit, Michigan, 48201, USA; ^5^ Use-inspired Biomaterials & Integrated Nano Delivery (U-BiND) Systems Laboratory Department of Pharmaceutical Sciences, Eugene Applebaum College of Pharmacy and Health Sciences, Wayne State University, Detroit, MI 48201, USA; ^6^ Department of Pharmaceutics and Pharmaceutical Technology, College of Pharmacy, Taif University, Taif 26571, Saudi Arabia; ^7^ Present address: Pharmaceutics Division, CSIR-Central Drug Research Institute, Lucknow 226031, India; ^8^ Present address: Cornell College, Mount Vernon, Iowa, 52314, USA; ^9^ Present Address: Department of Molecular Biology, Umeå University, 901 87 Umeå, Sweden

**Keywords:** RCC, CFM, CARP-1, everolimus, nano-micelles

## Abstract

Current treatments for Renal Cell Carcinoma (RCC) include a combination of surgery, targeted therapy, and immunotherapy. Emergence of resistant RCCs contributes to failure of drugs and poor prognosis, and thus warrants development of new and improved treatment options for RCCs. Here we generated and characterized RCC cells that are resistant to Everolimus, a frontline mToR-targeted therapy, and tested whether our novel class of CARP-1 functional mimetic (CFM) compounds inhibit parental and Everolimus-resistant RCC cells. CFMs inhibited RCC cell viability in a dose-dependent manner that was comparable to Everolimus treatments. The GI_50_ dose of Everolimus for parental A498 cells was ∼1.2μM while it was <0.02μM for the parental UOK262 and UOK268 cells. The GI_50_ dose for Everolimus-resistant A498, UOK262, and UOK268 cells were ≥10.0μM, 1.8-7.0μM, and 7.0-≥10.0μM, respectively. CFM-4 and its novel analog CFM-4.16 inhibited viabilities of Everolimus resistant RCC cells albeit CFM-4.16 was more effective than CFM-4. CFM-dependent loss of RCC cell viabilities was due in part to reduced cyclin B1 levels, activation of pro-apoptotic, stress-activated protein kinases (SAPKs), and apoptosis. CFM-4.16 suppressed growth of resistant RCC cells in three-dimensional suspension cultures. However, CFMs are hydrophobic and their intravenous administration and dose escalation for in-vivo studies remain challenging. In this study, we encapsulated CFM-4.16 in Vitamin-E TPGS-based- nanomicelles that resulted in its water-soluble formulation with higher CFM-4.16 loading (30% w/w). This CFM-4.16 formulation inhibited viability of parental and Everolimus-resistant RCC cells *in vitro*, and suppressed growth of parental A498 RCC-cell-derived xenografts in part by stimulating apoptosis. These findings portent promising therapeutic potential of CFM-4.16 for treatment of RCCs.

## INTRODUCTION

Renal cell carcinoma (RCC) is the most common type of kidney cancer [1]. In the United States, recent estimates indicated a total of 62,700 new cancer cases and 14,240 deaths from kidney and renal pelvis cancers in 2016 with increase in occurrence of RCCs in the coming years [2, 3]. RCCs comprise of different types of renal epithelial tumors that include most commonly occurring conventional (clear cell) renal-cell carcinomas (ccRCCs) followed by the papillary renal cell carcinomas, Chromophobe renal carcinoma, Oncocytoma, and Collecting-duct carcinoma [4]. Most RCCs seem to occur sporadically while 1-4% of the cases have an inherited predisposition. Mutations in the Von Hippel-Lindau (VHL) tumor suppressor occur frequently in RCCs. Recent discoveries revealed activating mutations in MET oncogene and inactivating mutation in Fumarate Hydratase (FH) gene in the hereditary papillary renal carcinoma (HPRC) and hereditary leiomyomatosis and renal-cell cancers (HLRCC), respectively. RCC is generally very difficult to treat as the cells are largely resistant to many conventional therapies [5]. Currently, surgery remains the best treatment option [4], although 20-30% of the patients progress to develop metastatic disease. FDA approved agents for treatment of metastatic RCC include tyrosine kinase inhibitors (TKIs) such as sorafenib and sunitinib and mammalian target of rapamycin (mTOR) inhibitors temsiorlimus and everolimus [5–7]. These targeted therapies result in improved clinical outcomes, and although everolimus is the first drug used as a secondary treatment option for resistant RCCs, patients ultimately develop resistance to targeted therapies that correlates with poor overall prognosis [6, 7].

CARP-1 (Cell cycle and apoptosis regulator 1, aka CCAR1) is a peri-nuclear phospho-protein and a regulator of cell growth and apoptosis signaling [8–11]. CARP-1 not only functions as a transcriptional co-activator of steroid family of nuclear receptors and a regulator of adipogenesis through the glucocorticoid receptor (GR), it also regulates Adriamycin (ADR)-dependent apoptosis in part through co-activation of p53 [12, 13]. CARP-1 expression is often elevated in cells experiencing stress due to growth factor withdrawal or chemotherapy-induced cell cycle arrest and apoptosis [8, 9, 12]. Knockdown of CARP-1 resulted in resistance to apoptosis by ADR or EGFR tyrosine kinase inhibitors demonstrating requirement of CARP-1 in cell growth inhibitory and apoptosis signaling by these agents [8, 9, 12].

We previously discovered that CARP-1 also functions as a co-activator of the APC/C E3 ligase [10]. APC/C is a multi-subunit ubiquitin E3 ligase protein that plays a distinct role in cell cycle transitions. Misregulation of APC/C substrates such as Securin, Polo-like kinase (Plk) correlate with tumor progression [14, 15]. A chemical biology-based high-throughput screening of a chemical library resulted in identification of a number of novel, small molecule inhibitors (SMIs) of CARP-1 binding with APC/C subunit APC2 [10]. These compounds, termed CARP-1 functional mimetics (CFMs), block its interaction with APC2, cause G2M cell cycle arrest, and inhibit cell growth by inducing apoptosis in various cancer types [10, 11]. CFMs however have poor aqueous solubility.

The rapid advancement of nanotechnology allows alternative approaches to overcome limitations of conventional anti-cancer therapy. Drug encapsulation, using polymeric nanoparticles to advance their transport to the cancer site, has become a recent standard in novel anti-cancer methods [16–19]. In effect, the use of non-toxic and biocompatible nanoparticles for formulations of anti-neoplastic agents results in improved pharmacokinetics and specificity of the anti-cancer agents. The nanoparticle-based formulations circumvent the poor aqueous solubility of many compounds and help improve the outcomes of chemotherapy. The amphiphilic nanocarrier D-alpha-tocopheryl polyethylene glycol succinate (Vitamin E TPGS), is an excellent candidate for encapsulation of hydrophobic drugs. The TPGS nano-micelles thereby improve the solubility, stability and bioavailability of the loaded lipophilic drug, while the TPGS by itself inhibits P-glycoprotein MDR to enhance anticancer effect of loaded agent [20–22]. Addition of low molecular weight styrene-maleic acid (SMA) polymer to generate SMA-TPGS block co-polymer facilitates formation of nano-micelles. These nano-micelles have high drug loading, good water solubility, nontoxicity, and biosafety profiles for use *in vivo* delivery of drug payload [23]. In this regard, the native SMA polymer conjugated to neocarzinostatin (SMANCS) was approved for human use [24–25].

Here we investigated (a) the molecular mechanisms of RCC cell growth inhibition by the CFM compounds, (b) the extent to which these compounds inhibit growth of drug (Everolimus)-resistant RCC cells, and (c) whether the SMA-TPGS nano-formulation of CFM-4.16 circumvents the solubility concerns of CFM compounds to permit its intravenous administration in conducting *in vivo* studies. Our data indicate that CFMs inhibit growth of parental as well as Everolimus-resistant RCC cells in part by promoting apoptosis. The TPGS-based nano-formulation of CFM-4.16 inhibits viability of RCC cells *in vitro* and their growth as xenografted tumors in immunocompromised mice.

## RESULTS

### CFMs inhibit viabilities of RCC cells

**Figure 1:**
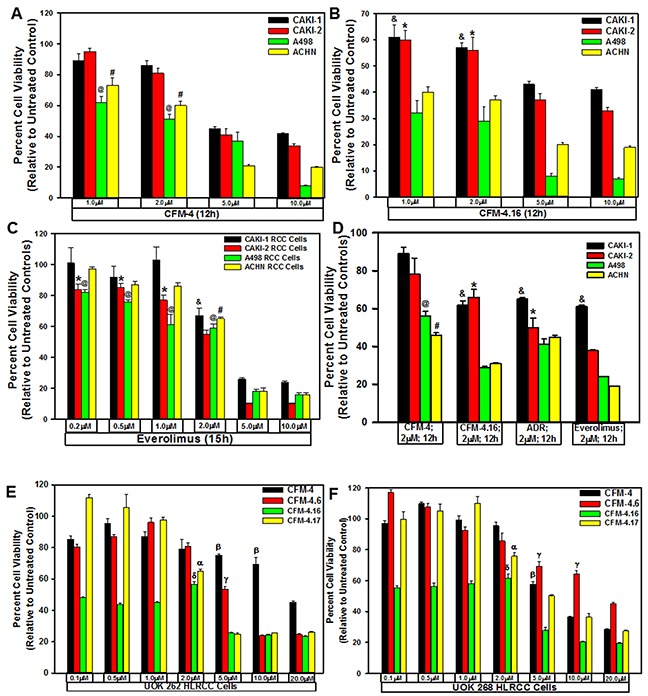
CFMs inhibit RCC cell growth We treated noted cell lines either with DMSO (Control), with various CFMs **(A, B, D-F)**, Everolimus **(C)**, or ADR (D) for indicated dose and time. We determined cell viability by MTT assay. The data in the histograms represent means of three independent experiments with 4-6 replicates for each treatment; bars, S.E. A-D, @,#,&,^*^, E-F, α,β,γ,δ, statistically significant inhibition (p = <0.05) relative to DMSO-treated respective controls.

Our prior findings had indicated anti-cancer properties of a novel class of CFM compounds [10], and our recent medicinal chemistry-based structure-activity relationship (SAR) studies reported identification of CFM analogs, in particular CFM-4.16, that was a superior inhibitor of parental and drug-resistant human and murine triple-negative breast cancer cells *in vitro* and *in vivo* [26]. Since emergence of resistance to current therapeutics remains a formidable problem in effective treatment and management of RCCs in clinic [5–7], we speculated whether CFM class of compounds would be effective inhibitors of RCC cells and to the extent, these compounds would be suitable to inhibit the resistant RCCs. We tested this possibility by conducting studies as detailed below. First, we evaluated potencies of the parent compound CFM-4 and its analogs CFM-4.6, −4.16, and −4.17 in cell culture studies utilizing RCC cell lines of ccRCC (CAKI-1, A498), papillary RCC (ACHN, CAKI-2), and HLRCC (UOK 262 and UOK 268) origins [27] by MTT based assays. As shown in Figure 1, CFM-4.16 dose of 1.0 and 2.0 μM over a period of 12h caused a greater loss of viability of all the RCC cells when compared to the RCC cells treated with similar doses of CFM-4 compound. Since Everolimus is one of the currently used targeted therapy for RCCs, we tested whether Everolimus treatments also provoked loss of viabilities of the RCC cells and to the extent anti-RCC effects of Everolimus were different from the CFM-4.16 treatments. The Everolimus doses of 0.2, 0.5, 1.0, and 2.0μM caused a moderate 20-40% loss in the viabilities of RCC cells, the doses of 5.0 and 10.0μM however provoked a greater than 60-70% reduction in the viabilities of the RCC cells (Figure 1C). Given that the molecular masses of Everolimus, Doxorubicin, and CFM-4.16 are 958.22, 543.5, and 440.35, respectively, a 1μM dose of Everolimus will have an approximate molar equivalence to a 2.0μM dose of either Doxorubicin or CFM-4.16. Thus although treatments with 5.0 or 10.0μM doses of Everolimus, CFM-4, and CFM-4.16 provoked a similar 60-80% reduction in viabilities of the RCC cells, a 2.0μM dose of CFM-4.16 induced a 40-60% loss of RCC cell viabilities (Figure 1B) while a 1μM dose of Everolimus caused a moderate 20-40% reduction in RCC cell viabilities (Figure 1C). These data in Figure 1 suggest that the RCC cells are likely more sensitive to inhibition by CFM-4.16 when compared with CFM-4 or Everolimus at the equivalent doses of up to 2μM of each compound. Additional dose response studies with reference to A498, CAKI-1, and ACHN RCC cells revealed that CFM-4.16 dose for inhibition of the cell growth by 50% (GI_50_) was ∼1.5-1.8μM, its dose for inducing a 50% cytotoxic effects (LC_50_) was 5.5-5.7μM (not shown). Further cell viability-based assays revealed that ∼10-fold higher dose of CFM-4.16 was required for a 50% cell growth inhibition of the renal epithelial HEK293 and HK2 cells when compared with the RCC UOK262 and A498 cells (Supplementary Figure 1).

**Figure 2:**
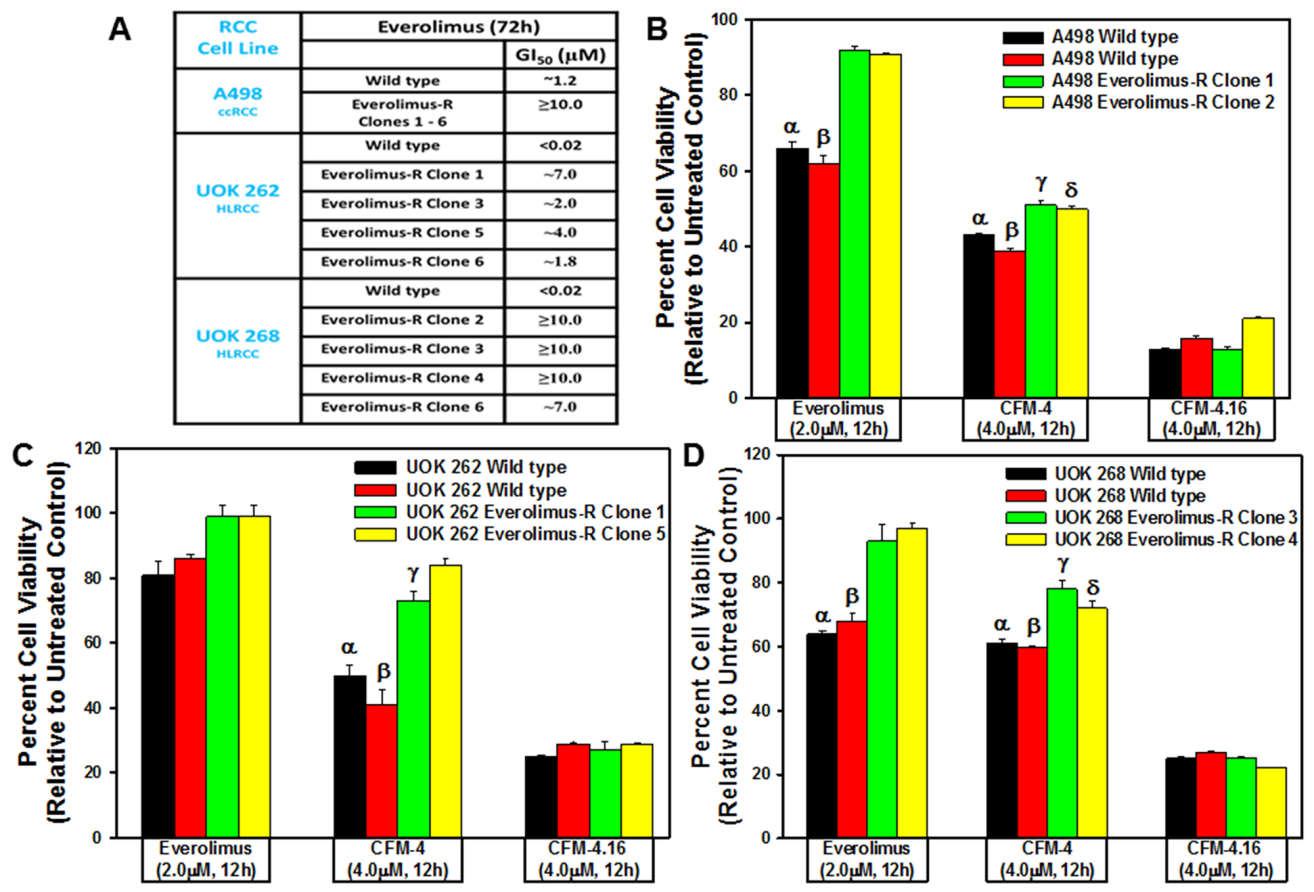
CFM-4. 16 inhibits Everolimus-resistant RCC cell growth **(A)** GI_50_ values of parental and drug-resistant RCC cells. In the case of Everolimus-resistant cells, the respective parental and resistant sublines were treated with 0.02, 0.1, 0.2, 1.0, 2.0, 4.0, and 10.0 μM dose of Everolimus. Percent cell viabilities were determined relative to respective DMSO-treated controls. The data in the GI_50_ columns represent means of three independent experiments. **(B-D)** Indicated parental and their respective drug resistant RCC cells were either untreated (Control) or treated with noted doses of Everolimus, CFM-4, or CFM-4.16 for 12h. We determined cell viability by MTT assay. The histogram columns represent means of three independent experiments with 4-6 replicates for each treatment; bars, S.E. B-D, α,β,γ,δ, statistically significant inhibition (p = <0.05) relative to DMSO-treated respective controls.

To test whether CFM class of compounds are also effective inhibitors of the resistant RCCs, as a proof-of-concept strategy, we first generated and characterized a number of RCC sublines that were resistant to Everolimus. We cultured A498, UOK 262, and UOK 268 RCC cells in the chronic presence of escalating doses of Everolimus until the resistance emerged. As shown in Figure 2A, the GI_50_ doses for Everolimus were 1.2, 0.02, and 0.02μM for the parental A498, UOK 262 and UOK 268 RCC cells, respectively. However, for the Everolimus-resistant sublines of A498 and UOK 268 origin, the GI_50_ dose of Everolimus was ≥10.0μM with the exception of Everolimus-resistant UOK 268 clone 6 subline that had the GI_50_ dose of ∼7.0μM. Interestingly, the Everolimus resistant sublines of UOK 262 origin had a variable GI_50_ doses with ∼7.0, ∼2.0, ∼4.0, and ∼1.8μM for clone 1, 3, 5, and 6 sublines, respectively. These data in Figure 2A strongly suggest that all the RCC cells developed a robust level of resistance to Everolimus. We next investigated whether the CFM compounds inhibited viabilities of the Everolimus resistant RCC cells by conducting MTT based assays as in Figure 1. As shown in Figure 2B-2D, a 4.0μM dose of CFM-4 caused ∼50% loss of viability of the Everolimus resistant A498 cells while a moderate ∼20% reduction in the viability of the Everolimus resistant UOK 262 and UOK 268 cells was noted. A 4.0μM dose of CFM-4.16 on the other hand provoked ∼80% reduction in the viabilities of all the Everolimus-resistant RCC cells. These data corroborate our current findings in Figure 1 and our recent studies demonstrating increased effectiveness of CFM-4.16 in attenuating viabilities of the parental RCC cells as well as drug-resistant RCC (Figure 2) and TNBC cells [26].

### CFM-4.16 Stimulates apoptosis in parental and resistant RCC cells by activating p38 MAP kinase, c-Jun N-terminal kinase (JNK) and enhancing expression of CCAR-1/CARP-1

**Figure 3:**
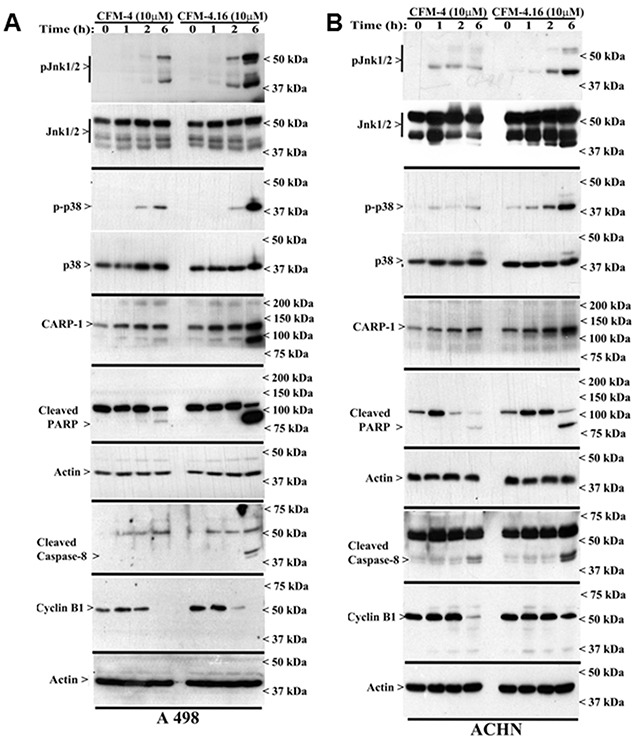

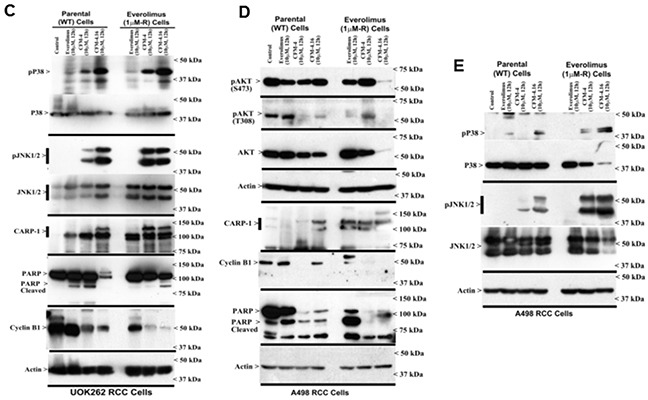
CFM-4. 16 stimulates apoptosis in parental and Everolimus-resistant RCC cells in part by upregulating pro-apoptotic CARP-1 and activating SAPKs **(A, B)** Indicated RCC cells were either untreated (Control, denoted as 0), treated with CFM-4, or CFM-4.16 for noted dose and time. We analyzed the cell lysates by western blotting (WB) as in Methods for levels of CARP-1, cyclin B1, cleaved PARP and caspase-8, and activation (phosphorylation) of pro-apoptotic p38 and JNK1/2 SAPKs. **(C-E)** Parental or Everolimus-resistant RCC cells were either untreated (Control), treated with Everolimus, CFM-4, or CFM-4.16 for noted dose and time. Cell lysates were analyzed by Western blotting (WB) for expression and/or activation of Akt, SAPKs, Cyclin B1, PARP, and CARP-1. The western blot membranes in panels A-E were probed with anti-actin antibodies to assess protein loading.

CARP-1 is a key transducer of apoptosis signaling by therapeutics such as Doxorubicin, Etoposide, and Gefitinib [8, 9], and CARP-1 expression was necessary for transduction of apoptotic/inhibitory signaling by these therapeutics as well as by our experimental CFM class of compounds in parental and drug-resistant TNBC cells [26]. Since CFM-4.16 was more effective in reducing viabilities of parental as well as Everolimus resistant RCC cells (Figure 2), we next investigated molecular mechanisms of apoptosis by CFM compounds and to the extent CARP-1 was required for inhibition of RCC cells by CFMs. Our western blot (WB) analyses revealed that equimolar (10μM) dose of CFM-4 or CFM-4.16 stimulated CARP-1 expression and activation of pro-apoptotic, stress-activated protein kinases (SAPKs) in the RCC cells in a time-dependent manner (Figure 3A–3E). Although activation of both the p38α/β and JNK1/2 SAPKs occurred as early as 1h of treatment with CFMs, CFM-4.16 caused a rather robust activation of these SAPKs at 6h treatment period (Figure 3A, 3B). Co-incident with the SAPK activation, treatments with CFMs over a 6-12h period stimulated activation of PARP and caspase 8, while causing a significant decline in levels of mitotic cyclin B1 in the RCC cells (Figure 3A–3D). Since CFMs inhibited viabilities of Everolimus-resistant RCC cells, we then determined whether CFMs provoked apoptosis in the Everolimus-resistant RCC cells as well. As shown in Figure 3C-3E, both the compounds stimulated CARP-1 expression, activation of SAPKs, PARP cleavage, and loss of cyclin B1 in the Everolimus-resistant UOK262 and A498 RCC cells. Here again, CFM-4.16 caused a generally higher increase in CARP-1 levels, activation of SAPKs, PARP-1 cleavage in parental or Everolimus-resistant RCC cells when compared with the cells that were treated with CFM-4. CFM-4.16 treatments also provoked a greater loss of cyclin B1 in both the parental and Everolimus-resistant RCC cells when compared with their CFM-4-treated counterparts. These data suggest that CFMs inhibit RCC cell viabilities in part by promoting apoptosis.

In light of the fact that emergence of resistant RCC remain a significant clinical challenge, emerging evidence has indicated involvement of feed-back activation of PI3K-Akt node in patients treated with therapies targeting mToR complex [6]. Thus, inhibition of mTORC1 by agents such as Everolimus or Temsorlimus could potentially result in activation of mTORC2 to promote serine 473 phosphorylation and activation of Akt. Alternatively, inhibition of mTORC1 could also result in feed-back activation/stabilization of IRS-1 leading to activation of PI3K and subsequent phosphorylation of Akt (threonine 308). Our western-blot analysis revealed that CFM-4.16, but not CFM-4 or Everolimus, robustly inhibited expression as well as activation/phosphorylation of Akt in Everolimus-resistant A498 RCC cells (Figure 3D). These findings further support potential of CFM-4.16 to target drug-resistant RCCs.

**Figure 4:**
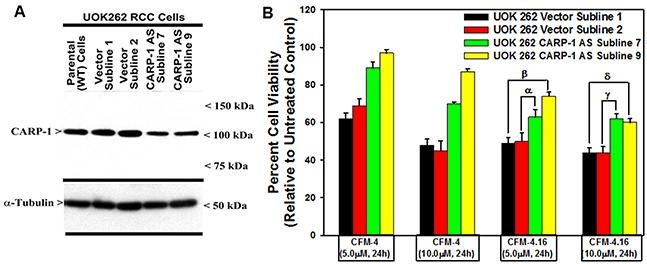
Knockdown of CARP-1 blocks CFM-4.16 effects **(A)** Cells were either untransfected, transfected with the pcDNA3/Hygro vector plasmid or plasmid expressing CARP-1 antisense, and stable, hygromycin-resistant cells were generated and characterized as detailed in methods. Cell lysates from indicated stable cell lines were subjected to WB analysis as in Figure 3 for levels of CARP-1 and α-tubulin. **(B)** The Indicated vector or CARP-1 antisense expressing RCC sublines were either untreated (Control) or treated with noted doses of CFM-4, or CFM-4.16 for 24h. Cell viability was determined by MTT assay. The histogram columns represent means of three independent experiments; bars, S.E. α, β, γ, δ, p = <0.05 relative to the vector expressing subline treated with CFM-4.16 only.

We next clarified whether the CFM compounds required CARP-1 to inhibit viabilities of the RCC cells. For this purpose, we first generated and characterized multiple, independent hygromycin-resistant stable sublines of UOK262 cells that express reduced CARP-1 as detailed in methods. The parental UOK262 cells were transfected with a plasmid encoding CARP-1 antisense or its vector counterpart, and transfected cells were cultured in chronic presence of hygromycin over a period of 6-8 weeks to obtain resistant sublines essentially following our previously described methods [8]. As shown in Figure 4A, stable expression of CARP-1 antisense plasmid caused reduced levels of CARP-1 in two sublines when compared with the levels of CARP-1 noted in two, vector-expressing sublines or the parental UOK262 cells. We then determined whether knock-down of CARP-1 expression interfered with loss of viabilities induced by treatments with CFM compounds. Our data in Figure 4B demonstrate that depletion of CARP-1 in the UOK262 cells resulted in a significant abrogation of inhibitory effects of both the CFM compounds when compared with their vector-expressing counterparts. Taken together, our findings in Figures 3 and 4 suggest that CFMs stimulate apoptosis in parental and resistant RCC cells and CARP-1 expression is required in transduction of inhibitory effects of the CFM compounds.

### CFM-4.16 suppresses three-dimensional growth of the parental and everolimus-resistant RCC cells

**Figure 5:**
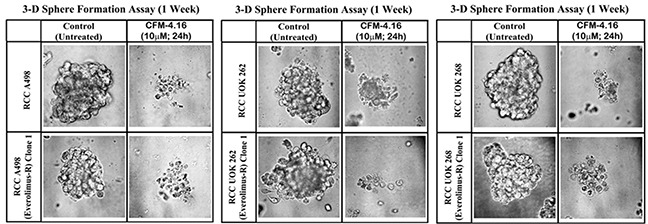
CFM-4.16 inhibits growth of RCC spheres derived from parental and Everolimus-resistant cells Parental and Everolimus-resistant RCC cells were grown as spheres as detailed in Methods. The sphere cultures were either untreated (Control) or treated with CFM-4.16 for noted dose and time. The untreated and treated spheres were then photographed as in methods. Representative photomicrographs of untreated and CFM-4.16 treated spheres are shown.

Recent studies have revealed culture of the RCC cells in a three-dimensional (3D) system as spheroids, and that the overall gene expression patterns of RCC spheroids in 3D more closely mimicked those observed in RCC tumors *in vivo* [28]. These studies further suggested suitability of 3D RCC spheroids from established cell lines as well as patient-derived, primary RCC tumors for pharmacological testing and investigating molecular mechanisms of RCC metastasis. Since CFM-4.16 inhibited growth of mammospheres derived from parental and drug-resistant human TNBC cells [26], we tested whether CFM-4.16 will inhibit growth of the RCC spheroids in 3D culture conditions. As shown in Figure 5, the parental A498, UOK262, and UOK268 RCC cells as well as their respective, Everolimus-resistant sublines formed RCC spheres in 3D culture conditions that are detailed in methods. Consistent with our observations with the human TNBC mammospheres, CFM-4.16 caused marked disintegration of spheres of parental and Everolimus resistant human RCC cells (Figure 5).

### Nanomicellar formulation of CFM-4.16 inhibits growth of parental and everolimus-resistant RCC cells *in vitro* and *in vivo* in part by stimulating apoptosis

The CFM compounds have poor aqueous solubility and consequent poor bioavailability for their use and development as potential anti-cancer agents. To address this issue, we previously generated and tested nano-lipid formulations (NLFs) of CFM-4 and CFM-4.16 compounds [26, 29]. These NLFs resulted in significant improvements in overall bioavailabilities of CFM-4 and CFM-4.16 when compared with the respective free compound [26, 29]. Here we generated nano-micellar formulations of CFM-4.16 and tested their abilities to inhibit growth of parental and resistant RCC cells *in vitro* and *in vivo*. As a first step, we synthesized, purified, and characterized a block copolymer (SMA-TPGS) as detailed in methods. The proton nuclear magnetic resonance spectroscopy (^1^H NMR) and Fourier transform infrared spectroscopy (FTIR) analysis revealed that the SMA-TPGS copolymer was a conjugate and not a physical mixture of TPGS with SMA (Supplementary Figures 2, 3). Next, we generated and characterized SMA-CFM-4.16 and SMA-TPGS-CFM-4.16 formulations. The mean diameter, the polydispersity index, and the Zeta potential of SMA-TPGS-CFM-4.16 formulation were 144.6nm ±20nm, 0.275 ±0.05, and −7.86 ±4 mV, respectively. The mean diameter, the polydispersity index, and the Zeta potential of SMA-CFM-4.16 formulation however were 123nm ±31nm, 0.163 ±0.07, and −18 ±5 mV, respectively. The slight increase in the particle size of the TPGS containing formulations is understandable, due to the hydrophilic PEG chains protruding out thereby increasing the hydrodynamic diameter. The critical micelles concentration (CMC) of the formulations was 0.010 and 0.023 mg/ml for SMA-TPGS-CFM-4.16 and SMA-CFM-4.16, respectively indicating high stability even on dilution of the sample. The Transmission Electron Microscopic (TEM) analyses did not indicate any morphological differences between CFM-4.16 loaded and unloaded nano-micelles (not shown). The loading of CFM-4.16 also had insignificant effect on the nano-micellar mean diameter, polydispersity index, or Zeta potential in comparison to the unloaded nano-micelles.

The type of polymer and the drug loading levels are critical factors that often influence drug release kinetics, cellular uptake and the therapeutic efficacy of the drug-loaded nanoparticles [30]. We then determined the encapsulation efficiency (EE) and drug loading content (DLC) for our formulations as detailed in methods. The EE and DLC for the SMA-CFM-4.16 was 77% and 17% respectively. The EE and DLC parameters for SMA-TPGS-CFM-4.16 preparation were 85.55 and 29%, respectively, suggesting improved loading due to the inclusion of emulsifier, TPGS. We next determined the stability of the formulations by their extended (2 months) storage at 4°C, 25°C, or 35°C with light protection. CFM-4.16 remained encapsulated in the SMA-TPGS nano-micelles with a recovery percentage of 99.73 ±1.10 at 4°C, 94.9 ±7.2 at 25°C, and 92.88 ±1.78 at 35°C. The recovery percentage of CFM-4.16 in SMA encapsulated formulation was 101.41 ±0.53 at 4°C, 96.42 ±0.42 at 25°C, and 90.81 ±1.32 at 35°C. Altogether, our results indicate that the CFM-4.16 micellar formulations have suitable drug loading and particle characteristics, and can be stored at 4°C or at room temperature (25°C). Based on this information, we proceeded to determine whether the CFM-4.16 nano-micellar formulations inhibit growth and viability of parental and drug-resistant RCC cells *in vitro* and *in vivo* as detailed below.

**Figure 6:**
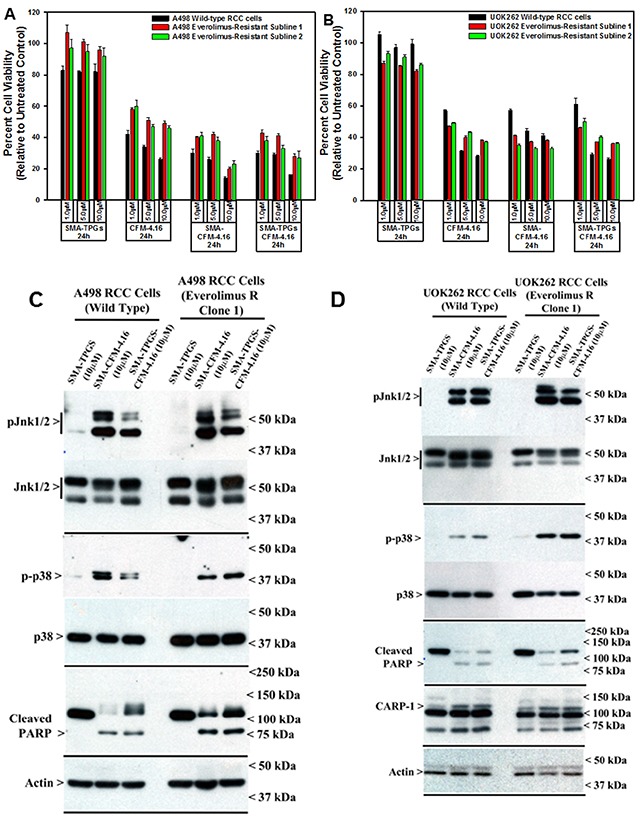
Nano-micellar formulations of CFM-4. 16 inhibits growth and stimulates apoptosis in parental and Everolimus-resistant RCC cells **(A, B)** Indicated RCC cells were either untreated (Control), treated with SMA-TPGS, CFM-4.16, SMA-CFM-4.16, or SMA-TPGS-CFM-4.16 for noted dose and time. (A, B) Cell viability was determined by MTT assay as in figure 2. The histogram columns represent means of three independent experiments with 4-6 replicates for each treatment; bars, S.E. **(C, D)** Cell lysates were analyzed by Western blotting (WB) as noted in Methods for levels of CARP-1, cleaved PARP and activation (phosphorylation) of pro-apoptotic p38, and JNK1/2 SAPKs essentially as in figure 3.

We treated the parental RCC cells and their respective, Everolimus-resistant sublines with various doses of block co-polymer (SMA-TPGS), free CFM-4.16, SMA-CFM-4.16, and SMA-TPGS-CFM-4.16 for 24h. The RCC cell viabilities were determined as described in methods. As shown in Figure 6A and 6B, the treatments of cells with various doses of block co-polymer alone elicited a very modest to no loss of their viabilities when compared with their untreated counterparts. The free CFM-4.16 or its nano-micellar formulations, on the other hand, inflicted a significant loss of viabilities of the parental as well as Everolimus-resistant RCC cells when compared with their respective, untreated counterparts. Of note is the fact that the free compound or its formulations at the three respective doses of each provoked a generally similar degree of reduction in RCC cell viabilities that ranged between 40-80%. A498 parental and resistant RCC cells albeit were more sensitive to the 10μM dose of either of the micellar formulations when compared with their CFM-4.16 treated counterparts, overall a similar range of reduction in the viabilities of cells that were treated with free compound or its micellar formulations would suggest for an excellent *in vitro* activity of CFM-4.16 formulations. Consistent with our data in Figure 3, the western blot analysis further revealed that treatments of parental or Everolimus-resistant RCC cells with 10μM dose of respective micellar formulations of CFM-4.16 also caused activation of pro-apoptotic SAPKs, P38α/β and JNK1/2, CARP-1 expression, and PARP cleavage when compared with their respective block co-polymer (SMA-TPGS)-treated cells (Figure 6C, 6D).

**Figure 7:**
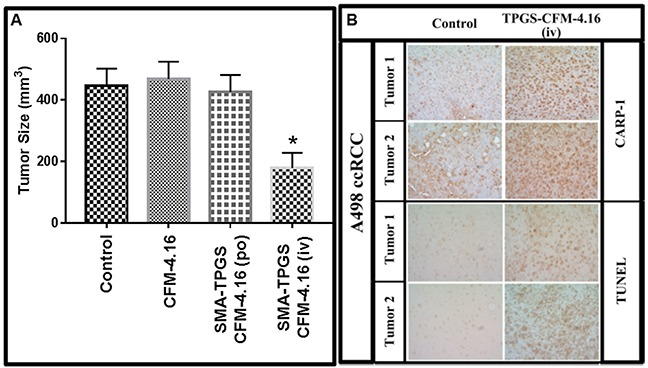
Nano-micellar formulation of CFM-4. 16 inhibits growth of RCC cell-derived xenografts **(A)** Histogram showing tumor size in the vehicle-treated (indicated as Control), CFM-4.16, or SMA-TPGS-CFM-4.16 (po or iv) treated, RCC (A498) xenograft-bearing animals. The xenograft establishment, treatment and analysis procedures were carried out essentially as detailed in Methods. The columns represent average values from a total of eight animals in respective group, bars, SE, significant where ^*^p = 0.05 vs Control. **(B)** SMA-TPGS-CFM-4.16 treatments (iv) induce CARP-1 expression and apoptosis in RCC tumor xenografts. Representative tumor tissues from two animals each from the vehicle-treated (noted as Control) or SMA-TPGS-CFM-4.16 treated groups were fixed in formalin, paraffin embedded, processed, and subjected to immuno-staining as detailed in Methods. Photomicrographs (400 x magnification) are shown for apoptosis (by TUNEL assay), and levels CARP-1 protein as noted in methods. Elevated apoptosis is indicated by increased brown staining or dark-brown spots in SMA-TPGS-CFM-4.16 panels stained with anti-CARP-1 antibodies or TUNEL, respectively.

We next examined the *in vivo* anti-tumor efficacy of nano-micellar formulation of CFM-4.16 (SMA-TPGS-CFM-4.16) in a highly aggressive RCC A498 orthotropic xenograft tumor bearing SCID mice as described in methods and our published protocols [26, 29]. In our previous studies, we prepared CFM-4.16 by dissolving it in 10% DMSO/cremophor plus sterile, distilled water with a pH of 4.5. We administered a dose of 30mg/kg/day of this preparation by intravenous (tail vein) injections for a total dose of 482mg/kg in SCID mice bearing human TNBC cell-derived xenografts. With the exception of a mild, <2% loss in body weight, the preparation did not cause any histological abnormalities in the treated animals, and lacked a therapeutic T/C values [26]. Consistent with our prior findings, intravenous (iv) administration of TPGS-CFM-4.16 or CFM-4.16 free drug provoked a mild loss in body weight (Supplementary Figure 4). On this basis, we chose a 30mg/kg/day dose of CFM-4.16 (free compound as DMSO/cremophor preparation or nano-micellar formulation) for use in current *in vivo* experiments. Although iv treatments with TPGS-CFM-4.16 elicited reduction in tumor size throughout the course of treatment (Supplementary Figure 5), iv administration of vehicle (Control) SMA-TPGS (total dose of 120mg/animal), DMSO/cremophor preparation of CFM-4.16 (total dose of 240mg/animal), or administration of SMA-TPGS-CFM-4.16 (total dose of 210mg/animal) by oral gavage failed to inhibit tumor growth (Figure 7A). However, only two i.v. injections of 30mg/kg/day of SMA-TPGS-CFM-4.16 (total dose of 60mg/animal) caused a significant reduction in tumor size when compared with the tumor sizes noted in the other treatment groups (Figure 7A). The blood samples from the treated animals were analyzed for presence of CFM-4.16 at the end of respective treatment. A 40-50μg/ml of CFM-4.16 was noted in mice that were treated with iv administration of CFM-4.16 free drug or TPGS-CFM-4.16 by oral gavage, while ∼3-fold higher levels of CFM-4.16 were noted in blood of animals that treated with iv administration of TPGS-CFM-4.16 formulation (Supplementary Figure 6). The HPLC analysis of the tumors from animals treated with i.v. injections of SMA-TPGS-CFM-4.16 revealed presence of CFM-4.16 in tumors (not shown). In addition, after the completion of the animal experiment, tumors from treatment and control groups were dissected, and cryosectioned for imaging of apoptotic signs using TUNEL (Terminal deoxynucleotidyl transferase dUTP nick end labeling) and CARP-1. The immuno-histological analysis of tumors from animals treated with i.v. injections of SMA-TPGS-CFM-4.16 showed elevated levels of CARP-1 and TUNEL-positive cells when compared with the tumors derived from the animals of control group (Figure 7B). Thus, the data suggest that nano-micellar formulation of CFM-4.16 enhance anti-tumor efficacy of CFM-4.16 when administered i.v. but not orally, at a significantly lower total dose when compared with the free compound.

## DISCUSSION

In an attempt to elucidate apoptosis signaling by chemotherapy, CARP-1 was identified as a perinuclear protein that was required for apoptosis by EGFR targeted therapeutics as well as DNA damaging agents such as adriamycin (ADR) and etoposide [8–10]. Although CARP-1 is a coactivator of steroid-thyroid receptor superfamily proteins [11, 12], we focused our efforts to exploit apoptosis signaling of CARP-1 for development of novel anti-cancer agents. Our high throughput chemical biology studies resulted in identification of small molecule compounds termed CARP-1 functional mimetics (CFMs) that bind with CARP-1 and function in part by stimulating apoptosis in various cancer cells [29, 31–33]. Further structure activity relationship (SAR) studies culminated in identification of CFM-4.16 that is structurally similar to the lead compound CFM-4. We recently reported that CFM-4.16 uniquely enhanced inhibition of TNBC cells only by the chemotherapeutic Adriamycin *in vitro*, while a nano-lipid formulation of CFM-4.16 in combination with Adriamycin was effective in suppressing growth of TNBC cell-derived tumors *in vivo* [26]. Here we initiated further studies to determine whether the CFM-4 and its analogs inhibit growth of RCC cells; and further explored molecular mechanism(s) of RCC cell death by these compounds. CFM-4.16 exposure resulted in a somewhat higher loss of RCC cell viability when compared with their loss of viabilities noted following treatments with CFM-4. It is therefore feasible that further rational medicinal chemistry modifications of these promising anticancer agents could yield additional novel small molecule compounds that may have greater potency and selectivity in inhibiting RCCs.

Approximately one third of RCC patients present metastases at diagnosis, and 30-70% of patients relapse within five years of surgery [5, 34]. During the last decade, several targeted therapies have been developed and approved for treatment of metastatic RCC. These second-line targeted therapies for the metastatic RCCs include mechanistic target of rapamycin (mToR) inhibitors such as Temsirolimus and Everolimus, and tyrosine kinase inhibitors such as Sunitinib and Sorafenib [35]. Although most of these therapies have substantially improved patient outcomes, none of these drugs are curative and resistance eventually develops. Here we utilized RCC cells of clear cell and hereditary leiomyomatosis subtypes to generate laboratory models of drug resistance by exposing them to chronic presence of Everolimus over an extended period. A number of Everolimus-resistant sublines of A498 (representative of ccRCC subtype), and UOK262 and UOK268 (representative of HLRCC subtype) were obtained and characterized for their resistance to Everolimus. In this proof-of-concept investigation, we find that CFM compounds are potent inhibitors of parental as well as Everolimus-resistant RCC cells. In fact, the compound CFM-4.16 seemed to be generally more effective inhibitor of these cells when compared with the parent compound CFM-4. Nevertheless, both the compounds function in part by stimulating apoptosis. Since CFMs function in part by binding with CARP-1 and interfere with activity of the Anaphase Promoting Complex/Cyclosome (APC/C) E3 ligase [10], our studies also revealed a CARP-1 requirement in transduction of growth inhibitory effects of CFM-4.16 in the RCC cells. At the molecular levels, CFMs target mitotic cyclin B1 and cause apoptosis in the parental and resistant RCC cells. This loss of cyclin B1 and stimulation of apoptosis by CFMs in RCC cells would be consistent with our prior findings demonstrating promotion of G2M cell cycle arrest, loss of mitotic cyclin B1, and apoptosis stimulation by CFMs in other cancer cell models [26, 29, 31–33].

Activity of the APC/C-CDC20 E3 ligase and levels of mitotic cyclin B1 are key regulators of G2M phase of cell cycle [36–38]. Many chemotherapeutic agents and experimental/medicinal compounds inhibit growth of a variety cancer cells in part by targeting cyclin B1 and promoting G2M arrest [39]. In this regard, a recent report highlighted targeting of mitotic cyclin B1 and promotion of G2M arrest by a plant-derived medicinal compound Sulforaphane (SFN) in the parental and Everolimus-resistant RCC cells of clear cell subtype [40]. Although efficacy of SFN was generally lower in the Everolimus-resistant RCC cells when compared with their parental counterparts, SFN did not promote early or late apoptosis. As noted above, we have found that CFMs attenuate cyclin B1 levels and promote G2M arrest in a variety of cancer cell types, and the fact that SFN caused G2M arrest in part by targeting Cyclin B1 it is likely that, similar to SFN, CFMs also stimulate G2M arrest in our parental and Everolimus-resistant RCC cells. One of the hypothesis of development of therapy resistance by cancer cells centers on the possibility that a small population of cancer cells that are able to resist elimination/clearance by apoptosis contribute to tumor dormancy and therapy resistance, and thus makes apoptosis a necessary attribute of an anticancer therapeutic [41]. It is therefore conceivable that agents such as SFN that are largely cyto-static may not necessarily be sufficient to overcome drug resistance. In this regard, our novel class of CFM scaffold that stimulate G2M arrest as well as cause apoptosis hold potential for therapeutic use for targeting drug-resistant cancers (current study and ref. 26).

CFM-4 and its analog CFM-4.16 have poor aqueous solubility of <1mg/ml. This attribute contributes to poor dissolution with consequent poor absorption and bioavailability. Recent advances in nano-formulation-based technology have allowed optimization of poorly soluble compounds for preclinical as well as clinical testing and use. In our prior proof-of-concept studies, nano-lipid formulations of CFM-4 and CFM-4.16 compounds were prepared by combining high-melting solid lipid carriers and vitamin E TPGS co-surfactant. These formulations were demonstrated to not only enhance solubility and oral bioavailability of the CFMs, but also were effective in suppressing NSCLC and TNBC cell-derived tumor growth in pre-clinical animal models when administered orally [26, 29]. Here we prepared a polymeric micelles based nano-drug delivery system of CFM-4.16 to improve its dissolution and bioavailability as reported with other nanoparticles published by our group [42–48]. A nanomicellar formulation of SMA-TPGS and CFM-4.16 resulted in improved dissolution and consequent enhanced absorption, which was also evident by the improved pharmacokinetic profile as previously reported [29]. Vitamin E-TPGS is FDA approved co-surfactant that acted as stabilizer and permeation enhancer while SMA contributed to good micellar property [17, 54, 55]. Thus, chemical conjugation of SMA and TPGS, namely SMA-TPGS creates more hydrophobic space, which provides higher CFM-4.16 encapsulation efficacy. Due to the crystalline nature of matrix lipids in solid particles, the space for the drug loading is often limited. Size and surface coating also play an important role in determining the mechanism and efficiency of nanoparticle. The particle size of ∼145 nm and surface charge is −7.86 mV of the CFM-4.16 loaded nanomicelle are optimal and safe for systemic (intravenous or oral) drug administration as well as competent for tumor delivery by passive targeting EPR-effect [49–51].

Our preliminary studies here indicate that both free and SMA-TPGS formulation of CFM-4.16 inhibit growth of RCC cells *in vitro*, in part by stimulating apoptosis. Consistent with our prior findings, CFM-4 or CFM-4.16 caused loss of cyclin B1, and upregulation of CARP-1, activation of p38 and JNK1/2 SAPKs, and cleavage of PARP in the RCC cells. Intravenous administration of the nano-micellar formulation of CFM-4.16 inhibited growth of RCC cell-derived tumor xenografts *in vivo* in part by stimulating CARP-1 levels and apoptosis. Taken together, our current data provide us with a further rationale to develop CFM compounds and their formulations for targeting parental and resistant RCCs.

## MATERIALS AND METHODS

### Cell culture, reagents and chemicals

Structure and synthesis of CFM-4, −4.16, and −4.17 compounds have been recently described [26], and their chemical structures are shown in the Supplementary Figure 7. A stock solution of 50mM of each CFM was prepared in dimethyl sulfoxide (DMSO) and stored at −20°C. Styrene maleic anhydride (SMA, MW 1600), D-alpha-tocopheryl polyethylene glycol succinate (Vitamin E-TPGS), and 3-[4,5-Dimethylthiazol-2-yl]-2,5diphenyltetrazolium bromide (MTT) were obtained from Sigma-Aldrich, St Louis, MO. Everolimus was purchased from SelleckChem, Boston, MA and a 50mM stock solution was prepared in DMSO and stored at −20°C, while clinical grade Adriamycin (ADR) was obtained from Karmanos Cancer Institute pharmacy, Detroit, MI. We purchased all other analytical grade reagents from Sigma-Aldrich (St Louis, MO) and used them without further purification.

DMEM, EMEM medium and antibiotics (penicillin and streptomycin) used in this study were purchased from Invitrogen Co. (Carlsbad, CA). Fetal bovine serum (FBS) and DMSO were obtained from Denville Scientific Inc. (Metuchen, NJ), and Fisher Scientific (Fair Lawn, NJ), respectively. The Protein Assay Kit was purchased from Bio-Rad Laboratories (Hercules, CA). The mouse monoclonal antibodies for β–actin were acquired from Sigma-Aldrich (St. Louis, MO). We purchased rabbit polyclonal antibodies for α-tubulin, Cyclin B1, Cleaved Caspase-8, PARP, phospho and total p38α/β, phospho- and total JNK1/2 SAPKs, and phospho and total Akt from Cell Signaling Technology (Beverly, MA). We have previously described generation and characterization of the anti-CARP-1 rabbit polyclonal antibodies [8].

The human RCC A498, CAKI-1, CAKI-2, and ACHN cells were from ATCC and kindly provided by Dr. Rajvir Dahiya (UCSF). The HLRCC (UOK 268 and UOK 262) cells were kindly provided by Dr. Marsten Lanehan (NCI). All the cells were routinely maintained as described before [52, 53]. All the cell culture media were supplemented with 10% FBS, 100 units/ml of penicillin, and 100 μg/ml of streptomycin, and the cells were kept at 37°C and 5% CO_2_. For cell growth and MTT studies, the cells were cultured in fresh media with 10% FBS prior to their treatments with various agents.

### Generation of everolimus-resistant RCC cells

The human RCC A498, UOK262, and UOK268 cells were cultured in the chronic presence (>6 months) of Everolimus. The parental A498 cells were initially treated with 500nM Everolimus for 3-4 weeks, followed by escalation to 1.0, 2.0, 4.0 and 10.0μM doses. The cells were cultured in continuous presence of each of the dose for 3-4 weeks till resistance developed and cells became adapted to growth in 2μM Everolimus. In the case of UOK262 and 268 RCC cells, the parental cells were initially cultured in 10nM Everolimus for 3-4 weeks. For selection of the resistant cells, everolimus dose was escalated to 20, 50, 100, 200, 500, 1000, and 2000nM. The UOK cells were cultured in continuous presence of each of the dose for 2-3 weeks till resistance developed and cells adapted to growth in 2μM Everolimus. Subsequent, routine maintenance of the resistant cells in the presence of 2μM Everolimus was continued and multiple, resistant sublines for each of the RCC cells were isolated and characterized for their growth inhibitory (GI_50_) dose of Everolimus by the MTT-based viability assays as detailed below.

### Generation of CARP-1 knock-down RCC cells

The human RCC UOK262 parental cells were transfected with vector plasmid pcDNA3/hygro or plasmid expressing CARP-1 anti-sense (Clone 1.6, ref 8). Multiple, stable sublines for hygromycin resistance were selected in the presence of 400μg/ml hygromycin (#10687010, Invitrogen Inc) following methods described before [8]. We determined the levels of CARP-1 in the parental, and vector or CARP-1 antisense plasmid-transfected RCC cells and their viabilities in the presence of CFM compounds by western blot and MTT assays, respectively, as described below.

### Cell viability assays

The cytotoxicity of CFM-4, −4.6, −4.16, −4.17, Everolimus, ADR, SMA-TPGS co-polymer, SMA-CFM-4.16, SMA-TPGS-CFM-4.16 in the RCC cells (A498, UOK262, and UOK268) was assessed by MTT assay. First, we seeded 5 × 10^3^ cells in the 96-well plate in triplicate, allowed the cells to grow in fresh culture media for another 24h, and treated them with respective agents for the noted dose and time. Control cells were treated with 0.1% DMSO in culture medium. After treatment, we performed an MTT assay. Briefly, 20 μL of 1mg/ml of MTT was added to each well and cells were incubated for 2-4h at 37°C. MTT was removed, and the resulting formazan products were dissolved by adding 50μl DMSO/well followed by colorimetric analysis using a multi-label plate reader at 570 nm (Victor3; PerkinElmer, Wellesley, MA).

### Western blot analysis

For protein expression analysis, we conducted western blot experiments. The RCC cells were treated with DMSO/Vehicle (Control) or indicated dose and time of the noted compound, and were lysed to prepare protein extracts. Cells were harvested and lysed in RIPA buffer (50mM Tris-HCI, pH 8.0, 150mM sodium chloride, 1.0% NP-40, 0.5% sodium deoxycholate, 0.1% sodium dodecyl sulfate (SDS), and 0.1% of protease inhibitor cocktail) for 20 min at 4°C. The lysates were then centrifuged at 14,000 rpm at 4°C for 15 min to get rid of debris. We then determined the protein concentrations of whole cell lysates using the Protein Assay Kit. Supernatant proteins, 50μg from each sample, were separated by SDS-10% polyacrylamide gel electrophoresis (SDS-PAGE) and transferred to polyvinylidene difluoride (PVDF) membrane (Bio-Rad, Hercules, CA) by standard procedures. The membranes were hybridized with primary antibodies followed by incubation with appropriate secondary antibodies. The antibody-bound proteins were visualized by treatment with the chemiluminescence detection reagent (Amersham Biosciences) according to the manufacturer's instructions, followed by exposure to X-ray film (Kodak X-Omat). The same membranes were then re-probed with either the anti-β actin or anti-α tubulin antibody, which was used as an internal control for protein loading.

### SMA-TPGS synthesis and micellar nano-formulation fabrication

We first synthesized SMA-TPGS block copolymer (SMA-TPGS) by adding known amounts of TPGS in NaHCO_3_ buffer at pH 8.9 with fixed amounts of anhydrous SMA to permit its anhydride ring opening reaction with the alcohol group of TPGS. All unconjugated reagents were removed by ultrafiltration (Millipore TFF, Milford, MA) of the SMA-TPGS conjugate prior to its lyophilization. For Morphology, Transmission Electron Microscopy (TEM) of the nanoparticles was assessed using JEOL JEM-1000 instrument (JEOL Ltd, Tokyo, Japan). Then, the products obtained were stored in the freezer until further use. We characterized nano-micelles by proton nuclear magnetic resonance spectroscopy (^1^H NMR) and Fourier transform infrared spectroscopy (FTIR). The structure of the synthesized SMA-TPGS copolymer was detected by ^1^H NMR in D_2_O. The -CH protons and methyl protons of SMA segment had signals at 5.2 and 1.69 ppm, respectively. The -CH2 protons of PEO part of TPGS had the peak at 3.65 ppm. We noted the lower peaks in the aliphatic region that belong to various moieties of vitamin E tails (Supplementary Figure 2, and ref 21). The proper synthesis of the SMA-TPGS co-polymer was also confirmed by the FTIR analysis, and was not found to be a physical mixture of TPGS with SMA as all measurements indicated the absence of any free crystalline particles in nano-micelles preparation (Supplementary Figure 3). Both SMA and TPGS inhibited crystallization of CFM-4.16 during nano-micelles formulation. We then fabricated the CFM loaded micelles according to our earlier published protocols [18, 54–57], followed by characterization of micelles for size, charge, critical micelles concentration (CMC), and drug loading as below.

#### Particle size, and zeta potentials

The particle size and surface charge (zeta potential), measurements were performed using a Beckman Coulter Delsa Nano-C-DLS Particle analyzer (Miami, FL) equipped with a 658 nm He-Ne laser. For particle size, we suspended the nano-micelles in de-ionized (DI) water, and detected the scattered light at 165° angle. We then obtained the peak average histograms from the intensity, volume and number from 70 scans to calculate the average diameter of the particles. The zeta potentials were evaluated by measuring the electrophoretic mobility of the charged particles under an applied electric field.

#### The Loading efficiency of SMA-CFM nano-micelles

We evaluated the CFM-4.16 loading content percentage in SMA-TPGS nano-micelles by High Performance Liquid Chromatography (HPLC). First, a method for analyzing drug content was developed and validated according to ICH guidelines [58]. We measured the standard curve of CFM-4.16 in DMSO and its successive dilutions with mobile phase at 309 nm (λ _max_). The calibration curve was linear in the range of 50–50,000 ng/ml with a correlation coefficient (R2) = 0.9999. The loading efficiency of micelles was calculated by dissolving a known amount of nano-micelles directly in DMSO and further dilution of drugs with the mobile phase followed by determination of the absorbance at 309 nm with respect to the standard curve as described previously [29].

#### Drug encapsulation efficiency (EE)

Free drug (non-incorporated in the SMA-TPGS) was separated by ultrafiltration centrifugation technique. Briefly, 1 mL of CFM-4.16 and SMA-TPGS-CFM 4.16 colloidal solution were placed in the upper chamber of a centrifuge tube matched with an ultrafilter and centrifuged for 15 min at 4000 rpm. The total drug content in CFM-4.16 nano-formulation was determined as follows. Aliquots of 1mL formulation dispersion were diluted appropriately by ethanol to dissolve the TPGS-SMA ingredient, and the resulting suspension was then filtrated through 0.45*μ*m membrane filters. The filtered solution was analyzed by Waters® Alliance e2695 HPLC using Symmetry® C18 column (250mm × 4.6mm, 5*μ*m). The mobile phase was a mixture of Acetonitrile, Methanol, 10mM KH2PO4 buffer (65:20:15 v/v) with pH adjusted to 2, and the flow rate was maintained at 1.0mL/min. All the samples were analyzed at 309nm using empower PDA software. We then calculated the encapsulation efficiency (EE) and drug loading content (DLC) by the following equations:

$$\text{Drug loading content }(\text{DLC})=\frac{\begin{array}{l} \text{weight CFM}4.16\\ \text{encapsulated in micelles} \end{array}}{\begin{array}{l} \text{Total weight CFM}4.16\\ \text{loaded in micelles} \end{array}}\times 100$$ (1)

$$\text{Encapsulation Effeciency}(\text{EE})=\frac{\begin{array}{l} \text{Mass of CFM}4.16\\ \text{encapsulated in micelles} \end{array}}{\begin{array}{l} \text{Total mass of CFM}4.16\\ \text{Initially loaded in micelles} \end{array}}\times 100$$ (2)

### Three-dimensional renal sphere assays

The RCC cells were obtained from xenograft tumors derived from parental cells (see below) or from the parental and Everolimus-resistant RCC cells from a two-dimensional culture plate with ∼70-80% confluence. We performed the three-dimensional renal sphere cultures by essentially following the methods described by us before [26]. Briefly, the cells were washed twice in 1 x PBS and trypsinized following established protocols. We then pelleted the cells at 200 x g at room temperature, and re-suspended them in 5ml of sphere media (DMEM/F12 supplemented with 2mM L-glutamine, 100 U/ml penicillin, 100 U/ml streptomycin, 1 x B27 supplement, 20ng/ml recombinant human epidermal growth factor (EGF; Sigma), and 10ng/ml recombinant human basic fibroblast growth factor (bFGF; R&D Systems). We seeded ∼5000 viable cells per ml in an ultra-low adherent 60mm plate and incubated them at 37°C and 5% CO_2_ for two weeks without disturbing the plates. After the spheres formed, we added fresh media with or without 10μM CFM-4.16 and continued incubating cells for additional 24h at 37°C and 5% CO_2_. At the end of the incubation period, we photographed the spheres in the untreated and treated plates as described [59].

### Establishment of RCC cell-derived xenografts in immunocompromised mice

The experiments involving generation of RCC cell-derived sub-cutaneous xenografts were performed according to our previously published methods and protocols approved by the Institutional Laboratory Animal Care & Use Committee (IACUC) at the Wayne State University [26, 29]. Female, 5-weeks old NCR SCID mice with Lc background were purchased from Charles River Laboratories (Horsham, PA).

For subcutaneous (sc) tumor xenograft studies, we first determined maximal tolerated doses for CFM-4.16 (prepared in 10% DMSO/cremophor + distilled, sterile water, and pH adjusted to 4.5), SMA-TPGS co-polymer, SMA-TPGS-CFM-4.16, and SMA-CFM-4.16 preparations. The MTD for free CFM-4.16 (prepared in DMSO/cremophor) have been described before, and a 30mg/kg/day iv injection was judged safe while a total dose of 482mg/kg provoked a mild ataxia with some tail and leg twitching that resolved within 1-2 minutes. This dose/schedule of free CFM-4.16 produced a mild weight loss of 1.6% body weight by day 7 (recovery by day 18). No other histological abnormalities were noted [26]. A 30mg/kg/day dose of SMA-TPGS, was injected (iv) while a 30mg/kg/day of SMA-TPGS-CFM-4.16 was administered by oral gavage in two females, NCR SCID mice for 10 days. The animals did not show any signs of toxicity, discomfort, or any histological abnormalities. These observations indicate suitable toxicity profile of SMA-TPGS co-polymer and its CFM-4.16 formulation. However, the iv injections of a 30mg/kg were best tolerated when administered on alternate days. Accordingly, for the efficacy studies we chose to administer daily the block co-polymer by iv route while the micellear formulation with CFM-4.16 was administered by oral gavage. The iv administration of the CFM-4.16 micellar formulation was conducted on every alternate day.

For efficacy studies, after a suitable period of acclimation, we subcutaneously implanted a suspension of 1 × 10^6^ A498 RCC cells in 200μl of serum-free Hank's balanced salt solution in flanks of each animal using a 27-gauge needle [60]. Tumors were allowed to grow unperturbed for 10-14 days. When tumors became palpable (200 mm^3^), the mice were randomly assigned to treatment or control groups of eight animals each. Mice were treated with Control, PBS only, SMA-TPGS co-polymer (30mg/kg; iv), SMA-TPGS-CFM-4.16 formulation (30mg/kg/day) by oral gavage for 10 days. In the case of the group of mice treated with iv administration of SMA-TPGS-CFM-4.16 formulation (30mg/kg), only two injections were administered where the first dose was followed by the second dose on the alternate day. The tumor weight and volume were measured daily, and mice were observed for changes in weight and side effects. The end points for assessing antitumor activity consisted of tumor weight, tumor growth inhibition (%T/C), and tumor cell kill Log10. Tumor weight (mg) = (A X B2)/2 where A and B are the tumor length and width (in mm), respectively. Tumor growth inhibition (T/C) was the median tumor weight in the treated group (T) when the median tumor weight in the control group reached 750mg. Results were expressed as percentage. According to NCI-accepted criteria, a treatment is considered effective if T/C is < 42%. Tumor growth delay (T-C) is the difference between the median time (in days) required for the treatment group tumors (T) to reach 1000 mg and the median time (days) for the control group tumors to reach the same weight. The animals were sacrificed on day 10 and tumor tissues were collected immediately after tumor volume measurement. Tumor volumes were calculated by the modified ellipsoidal formula. Tumor volume = 1/2(length × width2). Representative tumor samples were stored at −80°C for subsequent analysis.

### Statistical analysis

The statistical analysis was done using Prism 6.0 software (Graph Pad Software Inc., San Diego, CA). The data were expressed as mean ± SEM and analyzed using a two-tailed Student t-test or one-way ANOVA followed by a post hoc test. A p value of <0.05 was considered statistically significant.

## SUPPLEMENTARY MATERIALS FIGURES



## References

[1] Siegel RL, Miller KD, Jemal A (2016). Cancer statistics, 2016. CA Cancer J Clin.

[2] Cohen HT, McGovern FJ (2005). Renal-cell carcinoma. N Engl J Med.

[3] Rini BI, Campbell SC, Escudier B (2009). Renal cell carcinoma. Lancet.

[4] Pavlovich CP, Schmidt LS (2004). Searching for the hereditary causes of renal-cell carcinoma. Nat Rev Cancer.

[5] Amato RJ (2000). Chemotherapy for renal cell carcinoma. Semin Oncol.

[6] Voss MH, Molina AM, Motzer RJ (2011). mTOR inhibitors in advanced renal cell carcinoma. Hematol Oncol Clin North Am.

[7] Minguet J, Smith KH, Bramlage CP, Bramlage P (2015). Targeted therapies for treatment of renal cell carcinoma: recent advances and future perspectives. Cancer Chemother Pharmacol.

[8] Rishi AK, Zhang L, Boyanapalli M, Wali A, Mohammad RM, Yu Y, Fontana JA, Hatfield JS, Dawson MI, Majumdar AP, Reichert U (2003). Identification and characterization of a cell cycle and apoptosis regulatory protein-1 as a novel mediator of apoptosis signaling by retinoid CD437. J Biol Chem.

[9] Rishi AK, Zhang L, Yu Y, Jiang Y, Nautiyal J, Wali A, Fontana JA, Levi E, Majumdar AP (2006). Cell cycle- and apoptosis-regulatory protein-1 is involved in apoptosis signaling by epidermal growth factor receptor. J Biol Chem.

[10] Puliyappadamba VT, Wu W, Bevis D, Zhang L, Polin L, Kilkuskie R, Finley RL, Larsen SD, Levi E, Miller FR, Wali A, Rishi AK (2011). Antagonists of anaphase-promoting complex (APC)-2-cell cycle and apoptosis regulatory protein (CARP)-1 interaction are novel regulators of cell growth and apoptosis. J Biol Chem.

[11] Muthu M, Cheriyan VT, Rishi AK (2015). CARP-1/CCAR1: a biphasic regulator of cancer cell growth and apoptosis. Oncotarget.

[12] Kim JH, Yang CK, Heo K, Roeder RG, An W, Stallcup MR (2008). CCAR1, a key regulator of mediator complex recruitment to nuclear receptor transcription complexes. Mol Cell.

[13] Ou CY, Chen TC, Lee JV, Wang JC, Stallcup MR (2014). Coregulator CCAR1 positively regulates adipocyte differentiation through the glucocorticoid signaling pathway. J Biol Chem.

[14] Harper JW, Burton JL, Solomon MJ (2002). The anaphase-promoting complex: it's not just for mitosis any more. Genes Dev.

[15] Lehman NL, Tibshirani R, Hsu JY, Natkunam Y, Harris BT, West RB, Masek MA, Montgomery K, van de Rijn M, Jackson PK (2007). Oncogenic regulators and substrates of the anaphase promoting complex/cyclosome are frequently overexpressed in malignant tumors. Am J Pathol.

[16] Kedar U, Phutane P, Shidhaye S, Kadam V (2010). Advances in polymeric micelles for drug delivery and tumor targeting. Nanomedicine (Lond).

[17] Wickens JM, Alsaab HO, Kesharwani P, Bhise K, Amin MC, Tekade RK, Gupta U, Iyer AK (2017). Recent advances in hyaluronic acid-decorated nanocarriers for targeted cancer therapy. Drug Discov Today.

[18] Luong D, Kesharwani P, Alsaab HO, Sau S, Padhye S, Sarkar FH, Iyer AK (2017). Folic acid conjugated polymeric micelles loaded with a curcumin difluorinated analog for targeting cervical and ovarian cancers. Colloids Surf B Biointerfaces.

[19] Alsaab H, Alzhrani RM, Kesharwani P, Sau S, Boddu SH, Iyer AK (2017). Folate decorated nanomicelles loaded with a potent curcumin analogue for targeting retinoblastoma. Pharmaceutics.

[20] Zhao L, Zhang Z, Bhakta G, Win KY, Dong Y, Chien S (2007). Chemotherapeutic engineering: vitamin E TPGS-emulsified nanoparticles of biodegradable polymers realized sustainable paclitaxel chemotherapy for 168 h *in vivo*. Chem Eng Sci.

[21] Mu L, Feng SS (2002). Vitamin E TPGS used as emulsifier in the solvent evaporation/extraction technique for fabrication of polymeric nanospheres for controlled release of paclitaxel (Taxol). J Control Release.

[22] Quin J, Engle D, Litwiller A, Peralta E, Grasch A, Boley T, Hazelrigg S (2005). Vitamin E succinate decreases lung cancer tumor growth in mice. J Surg Res.

[23] Greish K, Nagamitsu A, Fang J, Maeda H (2005). Copoly(styrene-maleic acid)-pirarubicin micelles: high tumor-targeting efficiency with little toxicity. Bioconjug Chem.

[24] Oda T, Maeda H (1987). Binding to and internalization by cultured cells of neocarzinostatin and enhancement of its actions by conjugation with lipophilic styrene-maleic acid copolymer. Cancer Res.

[25] Maeda H (2001). SMANCS and polymer-conjugated macromolecular drugs: advantages in cancer chemotherapy. Adv Drug Deliv Rev.

[26] Cheriyan VT, Muthu M, Patel K, Sekhar S, Rajeswaran W, Larsen SD, Polin L, Levi E, Singh M, Rishi AK (2016). CARP-1 functional mimetics are novel inhibitors of drug-resistant triple negative breast cancers. Oncotarget.

[27] Brodaczewska KK, Szczylik C, Fiedorowicz M, Porta C, Czarnecka AM (2016). Choosing the right cell line for renal cell cancer research. Mol Cancer.

[28] Pan T, Fong EL, Martinez M, Harrington DA, Lin SH, Farach-Carson MC, Satcher RL (2015). Three-dimensional (3D) culture of bone-derived human 786-O renal cell carcinoma retains relevant clinical characteristics of bone metastases. Cancer Lett.

[29] Muthu M, Somagoni J, Cheriyan VT, Munie S, Levi E, Ashour AE, Yassin AE, Alafeefy AM, Sochacki P, Polin LA, Reddy KB, Larsen SD, Singh M, Rishi AK (2015). Identification and testing of novel CARP-1 functional mimetic compounds as inhibitors of non-small cell lung and triple negative breast cancers. J Biomed Nanotechnol.

[30] Khadka P, Ro J, Kim H, Kim I, Kim JT, Kim H, Cho JM, Yun G, Lee J (2014). Pharmaceutical particle technologies: an approach to improve drug solubility, dissolution and bioavailability. Asian J Pharm Sci.

[31] Jamal S, Cheriyan VT, Muthu M, Munie S, Levi E, Ashour AE, Pass HI, Wali A, Singh M, Rishi AK (2014). CARP-1 functional mimetics are a novel class of small molecule inhibitors of malignant pleural mesothelioma cells. PLoS One.

[32] Muthu M, Cheriyan VT, Munie S, Levi E, Frank J, Ashour AE, Singh M, Rishi AK (2014). Mechanisms of neuroblastoma cell growth inhibition by CARP-1 functional mimetics. PLoS One.

[33] Ashour AE, Jamal S, Cheryan VT, Muthu M, Zoheir KM, Alafeefy AM, Abd-Allah AR, Levi E, Tarca AL, Polin LA, Rishi AK (2013). CARP-1 functional mimetics: a novel class of small molecule inhibitors of medulloblastoma cell growth. PLoS One.

[34] Kroeger N, Choueiri TK, Lee JL, Bjarnason GA, Knox JJ, MacKenzie MJ, Wood L, Srinivas S, Vaishamayan UN, Rha SY, Pal SK, Yuasa T, Donskov F (2014). Survival outcome and treatment response of patients with late relapse from renal cell carcinoma in the era of targeted therapy. Eur Urol.

[35] Von Klot CA, Merseburger AS, Kuczyk MA (2016). Novel therapeutic options for second-line therapy in metastatic renal cell carcinoma. Mol Clin Oncol.

[36] Izawa D, Pines J (2011). How APC/C-Cdc20 changes its substrate specificity in mitosis. Nat Cell Biol.

[37] Castro A, Bernis C, Vigneron S, Labbé JC, Lorca T (2005). The anaphase-promoting complex: a key factor in the regulation of cell cycle. Oncogene.

[38] Zhang J, Wan L, Dai X, Sun Y, Wei W (2014). Functional characterization of Anaphase Promoting Complex/Cyclosome (APC/C) E3 ubiquitin ligases in tumorigenesis. Biochim Biophys Acta.

[39] Yuan J, Yan R, Krämer A, Eckerdt F, Roller M, Kaufmann M, Strebhardt K (2004). Cyclin B1 depletion inhibits proliferation and induces apoptosis in human tumor cells. Oncogene.

[40] Juengel E, Maxeiner S, Rutz J, Justin S, Roos F, Khoder W, Tsaur I, Nelson K, Bechstein WO, Haferkamp A, Blaheta RA (2016). Sulforaphane inhibits proliferation and invasive activity of everolimus-resistant kidney cancer cells *in vitro*. Oncotarget.

[41] Wilson TR, Johnston PG, Longley DB (2009). Anti-apoptotic mechanisms of drug resistance in cancer. Curr Cancer Drug Targets.

[42] Sahu P, Kashaw SK, Jain S, Sau S, Iyer AK (2017). Assessment of penetration potential of pH responsive double walled biodegradable nanogels coated with eucalyptus oil for the controlled delivery of 5-fluorouracil: *in vitro* and *ex vivo* studies. J Control Release.

[43] Luong D, Kesharwani P, Killinger BA, Moszczynska A, Sarkar FH, Padhye S, Rishi AK, Iyer AK (2016). Solubility enhancement and targeted delivery of a potent anticancer flavonoid analogue to cancer cells using ligand decorated dendrimer nano-architectures. J Colloid Interface Sci.

[44] Luong D, Sau S, Kesharwani P, Iyer AK (2017). Polyvalent folate-dendrimer-coated iron oxide theranostic nanoparticles for simultaneous magnetic resonance imaging and precise cancer cell targeting. Biomacromolecules.

[45] Gawde KA, Kesharwani P, Sau S, Sarkar FH, Padhye S, Kashaw SK, Iyer AK (2017). Synthesis and characterization of folate decorated albumin bio-conjugate nanoparticles loaded with a synthetic curcumin difluorinated analogue. J Colloid Interface Sci.

[46] Amjad MW, Amin MC, Katas H, Butt AM, Kesharwani P, Iyer AK (2015). *In vivo* antitumor activity of folate-conjugated cholic acid-polyethylenimine micelles for the codelivery of doxorubicin and siRNA to colorectal adenocarcinomas. Mol Pharm.

[47] Kesharwani P, Xie L, Banerjee S, Mao G, Padhye S, Sarkar FH, Iyer AK (2015). Hyaluronic acid-conjugated polyamidoamine dendrimers for targeted delivery of 3,4-difluorobenzylidene curcumin to CD44 overexpressing pancreatic cancer cells. Colloids Surf B Biointerfaces.

[48] Kesharwani P, Banerjee S, Padhye S, Sarkar FH, Iyer AK (2015). Parenterally administrable nano-micelles of 3,4-difluorobenzylidene curcumin for treating pancreatic cancer. Colloids Surf B Biointerfaces.

[49] Iyer AK, Khaled G, Fang J, Maeda H (2006). Exploiting the enhanced permeability and retention effect for tumor targeting. Drug Discov Today.

[50] Amjad MW, Kesharwani P, Amin MC, Iyer AK (2017). Recent advances in the design, development, and targeting mechanisms of polymeric micelles for delivery of siRNA in cancer therapy. Prog Polym Sci.

[51] Greish K, Iyer AK, Fang J, Kawasuji M, Maeda H, Torchilin VP (2006). Enhanced permeability and retention (EPR) effect and tumor selective delivery of anticancer drugs. Delivery of Protein and Peptide Drugs in Cancer.

[52] Kawakami K, Yamamura S, Hirata H, Ueno K, Saini S, Majid S, Tanaka Y, Kawamoto K, Enokida H, Nakagawa M, Dahiya R (2011). Secreted frizzled-related protein-5 is epigenetically downregulated and functions as a tumor suppressor in kidney cancer. Int J Cancer.

[53] Yang Y, Lane AN, Ricketts CJ, Sourbier C, Wei MH, Shuch B, Pike L, Wu M, Rouault TA, Boros LG, Fan TW, Linehan WM (2013). Metabolic reprogramming for producing energy and reducing power in fumarate hydratase null cells from hereditary leiomyomatosis renal cell carcinoma. PLoS One.

[54] Iyer AK, Greish K, Fang J, Murakami R, Maeda H (2007). High-loading nanosized micelles of copoly(styrene-maleic acid)-zinc protoporphyrin for targeted delivery of a potent heme oxygenase inhibitor. Biomaterials.

[55] Iyer AK, Greish K, Seki T, Okazaki S, Fang J, Takeshita K, Maeda H (2007). Polymeric micelles of zinc protoporphyrin for tumor targeted delivery based on EPR effect and singlet oxygen generation. J Drug Target.

[56] Luong D, Sau S, Kesharwani P, Iyer AK (2017). Polyvalent folate-dendrimer-coated iron oxide theranostic nanoparticles for simultaneous magnetic resonance imaging and precise cancer cell targeting. Biomacromolecules.

[57] Kesharwani P, Banerjee S, Padhye S, Sarkar FH, Iyer AK (2015). Hyaluronic acid engineered nanomicelles loaded with 3,4-difluorobenzylidene curcumin for targeted killing of CD44+ stem-like pancreatic cancer cells. Biomacromolecules.

[58] Harmonised ICH Tripartite Guideline Validation of Analytical Procedures. Text and Methodology Q2(R1).

[59] Lombardo Y, de Giorgio A, Coombes CR, Stebbing J, Castellano L (2015). Mammosphere formation assay from human breast cancer tissues and cell lines. J Vis Exp.

[60] Sau S, Mondal SK, Kashaw SK, Iyer AK, Banerjee R (2017). Combination of cationic dexamethasone derivative and STAT3 inhibitor (WP1066) for aggressive melanoma: a strategy for repurposing a phase I clinical trial drug. Mol Cell Biochem.

