# Supplementary material for: A CARP-1 functional mimetic loaded vitamin E-TPGS micellar nano-formulation for inhibition of renal cell carcinoma
Source: Oncotarget. 2017 Sep 5;8(62):104928–45. doi: 10.18632/oncotarget.20650 (PMC5739610; doi:10.18632/oncotarget.20650)
Supplement: Supplementary file 1 [file oncotarget-08-104928-s001.pdf]

A CARP-1 functional mimetic loaded vitamin E-TPGS micellar nano-formulation for inhibition of Renal Cell Carcinoma

SUPPLEMENTARY MATERIALS

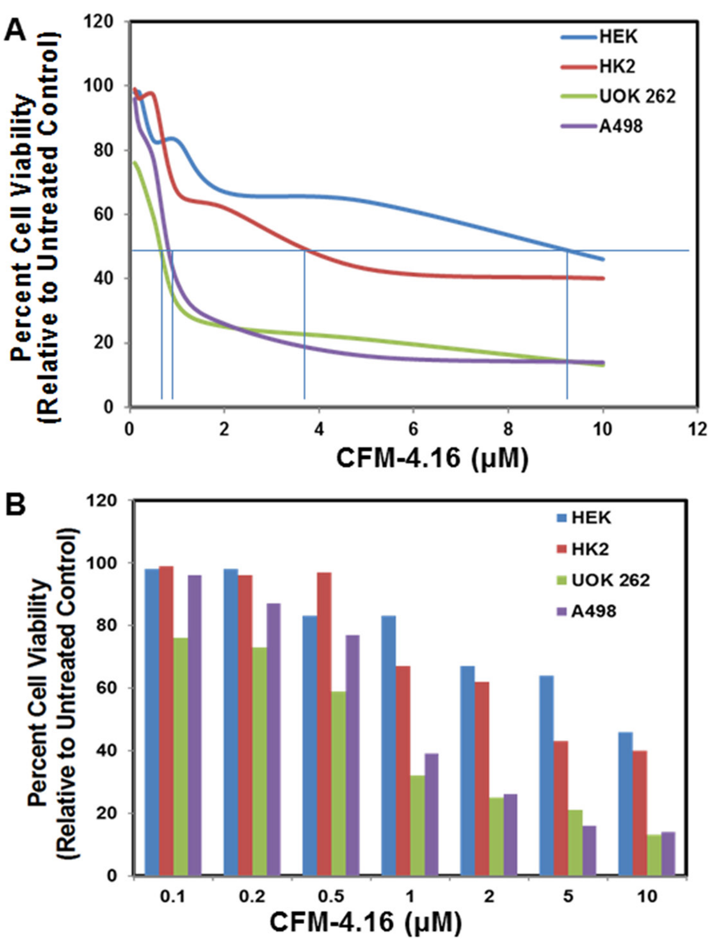

C

Cell Line	CFM-4.16, 72h, IC50 (µM)
UOK 262 Wild type	~0.85
A498 Wild type	~0.90
HEK 293 Wild Type	~9.2
HK2 Wild type	~3.8

Supplementary Figure 1: RCC cells are more sensitive to inhibition by CFM-4.16 when compared with the non-cancer renal epithelial cells. (A, B) Indicated cells were treated with 0.1, 0.2, 0.5, 1.0, 2.0, 5.0, and 10.0 µM dose of CFM-4.16. Percent cell viabilities were determined relative to respective DMSO-treated controls. The histogram (Lines in A or columns in B) represent means of two independent experiments with 4-6 replicates of each dose for the respective cell type **(C)** IC50 values of RCC and Renal epithelial cells treated with CFM-4.16 as in panels A and B.

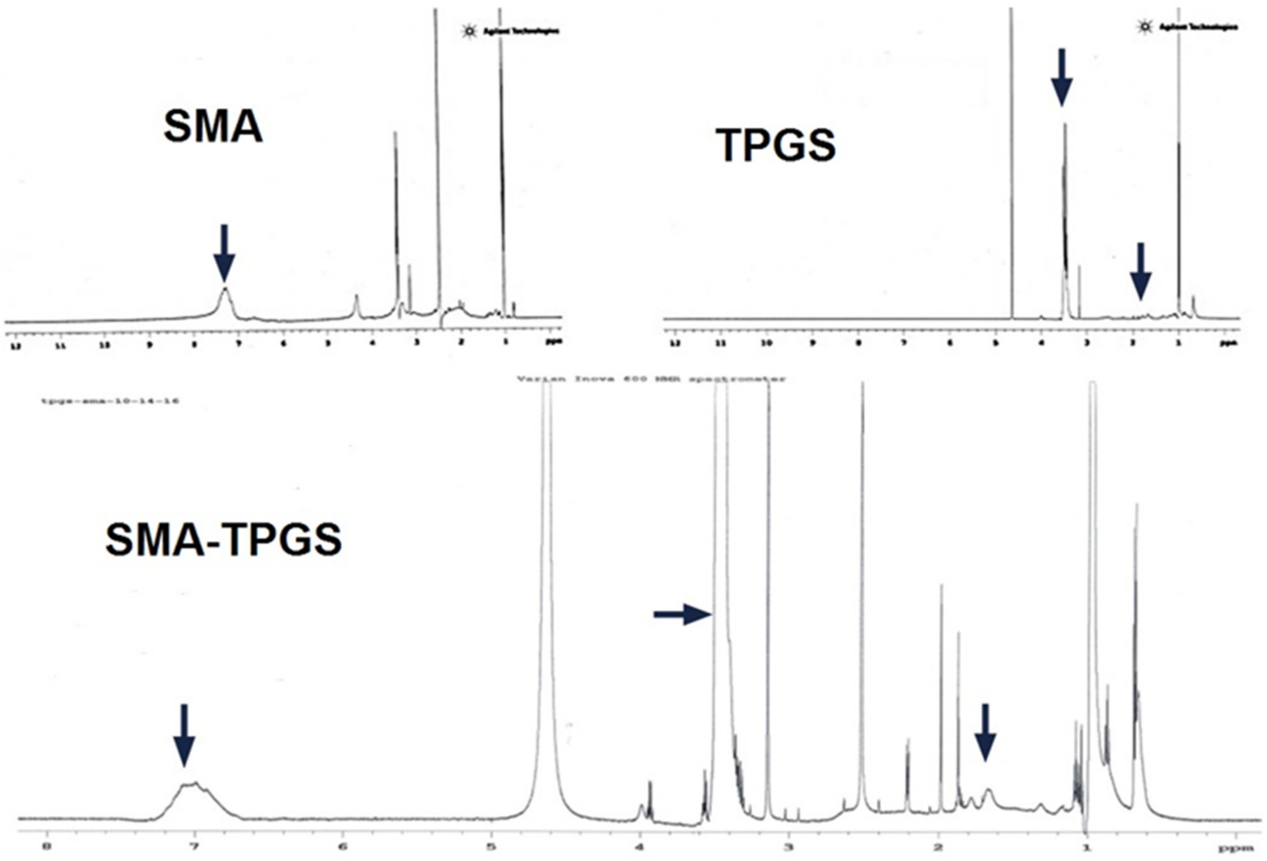

Supplementary Figure 2: H[1]-NMR profile for polymers used (SMA and TPGS) and the conjugate polymer (SMA-TPGS). The structure of the synthesized SMA-TPGS copolymer was detected by ^{1}H NMR in D_2O. The -CH protons and ring protons of SMA segment had signals at 1.69 ppm and 7.3 ppm, respectively. The-CH2 protons of PEO part of TPGS had the peak at 3.65 ppm. We noted the lower peaks in the aliphatic region that belong to various moieties of vitamin E tails. These peaks have been identified as well in the conjugate polymer as indicated by arrows.

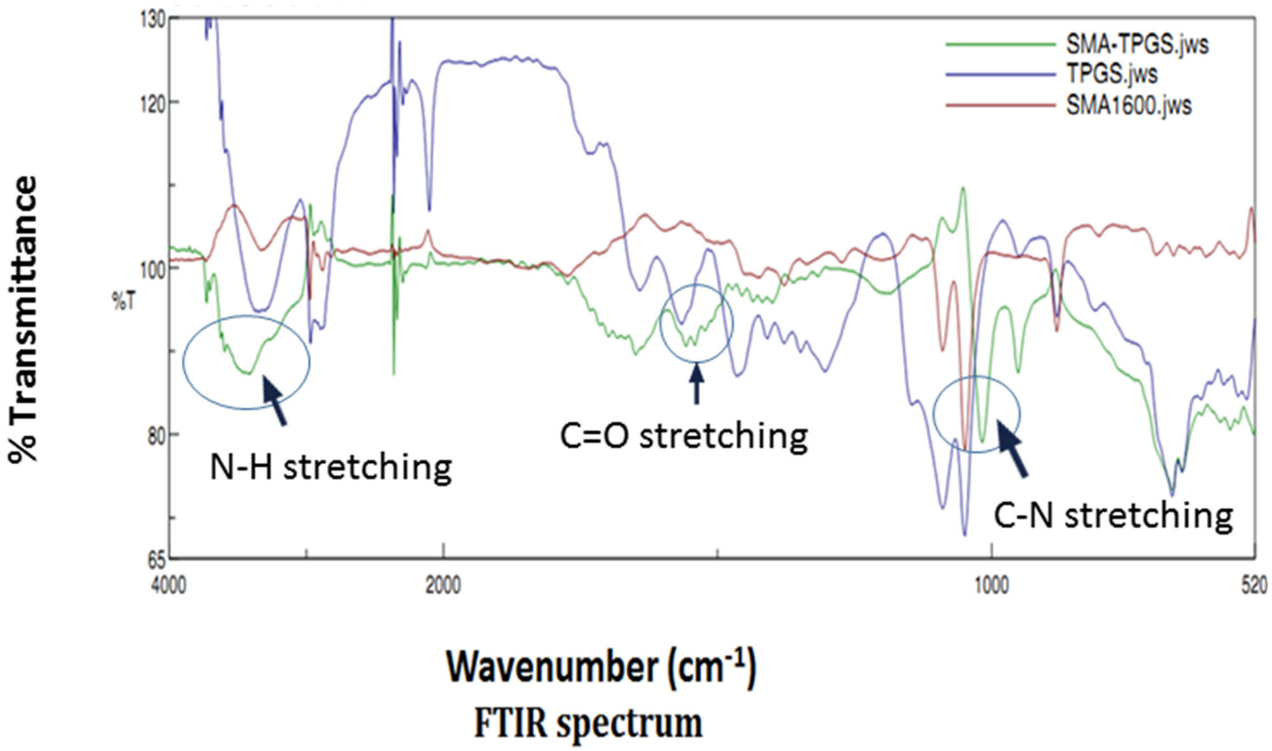

Supplementary Figure 3: FTIR data for polymers used (SMA and TPGS) and the conjugate polymer (SMA-TPGS). The arrows indicated forming an amide bond between the conjugate polymer (SMA-TPGS). Peaks were identified for C-N bond, C=O stretching, and N-H stretching at around 1100, 1640-1690, and 3100-3500 cm^{-1}, respectively.

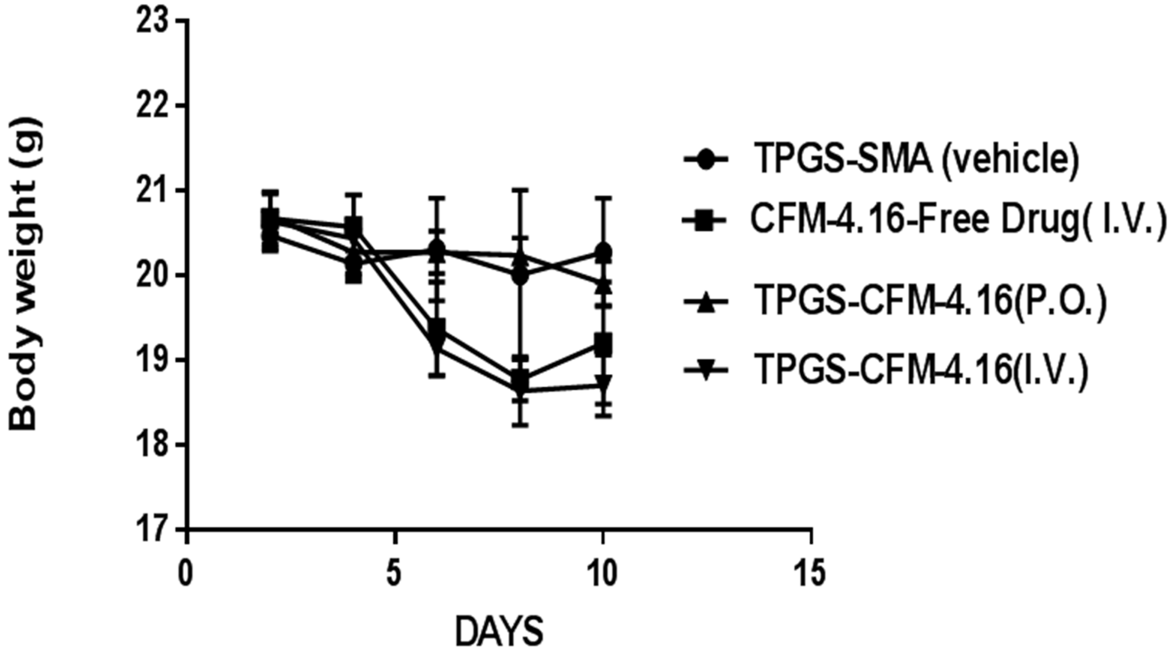

Supplementary Figure 4: Body weight changes of mice during treatment course. The lines represent average body weight from a total of eight animals in respective group that were measured on the indicated days; bars, SE.

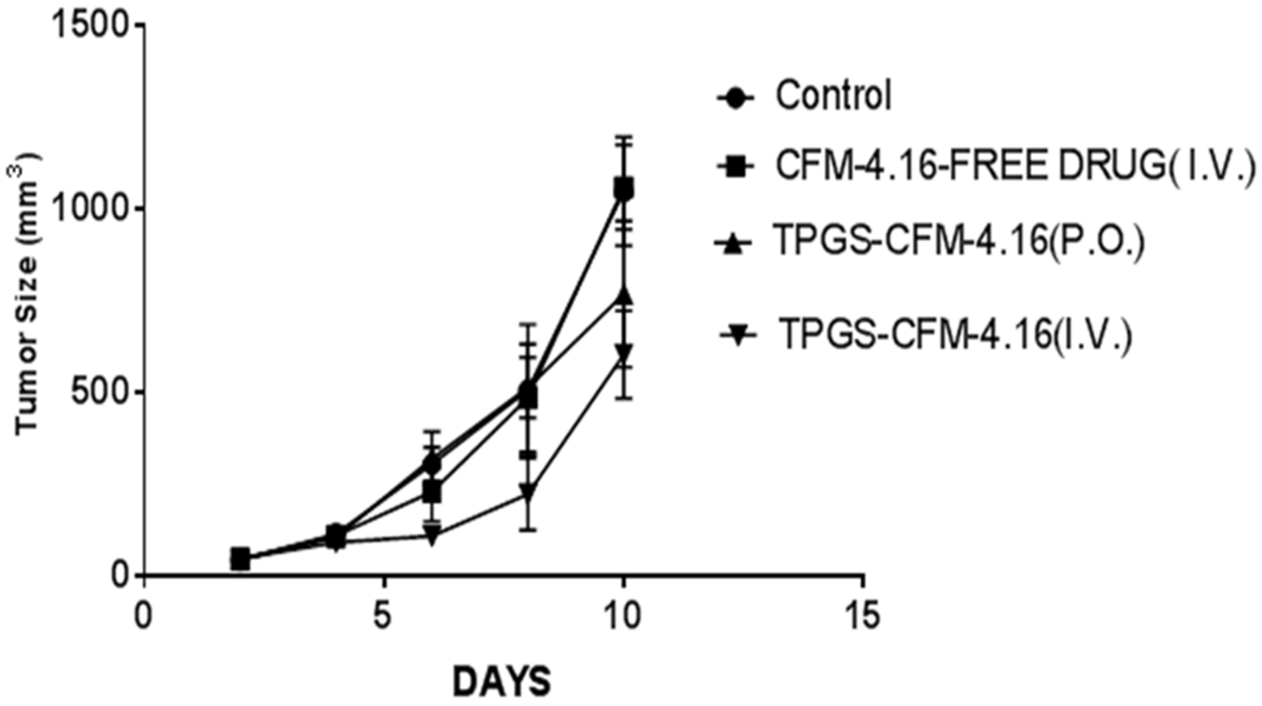

Supplementary Figure 5: Tumor size of mice during the course of treatment. The line represent average values from a total of eight animals in respective group, bars, SE.

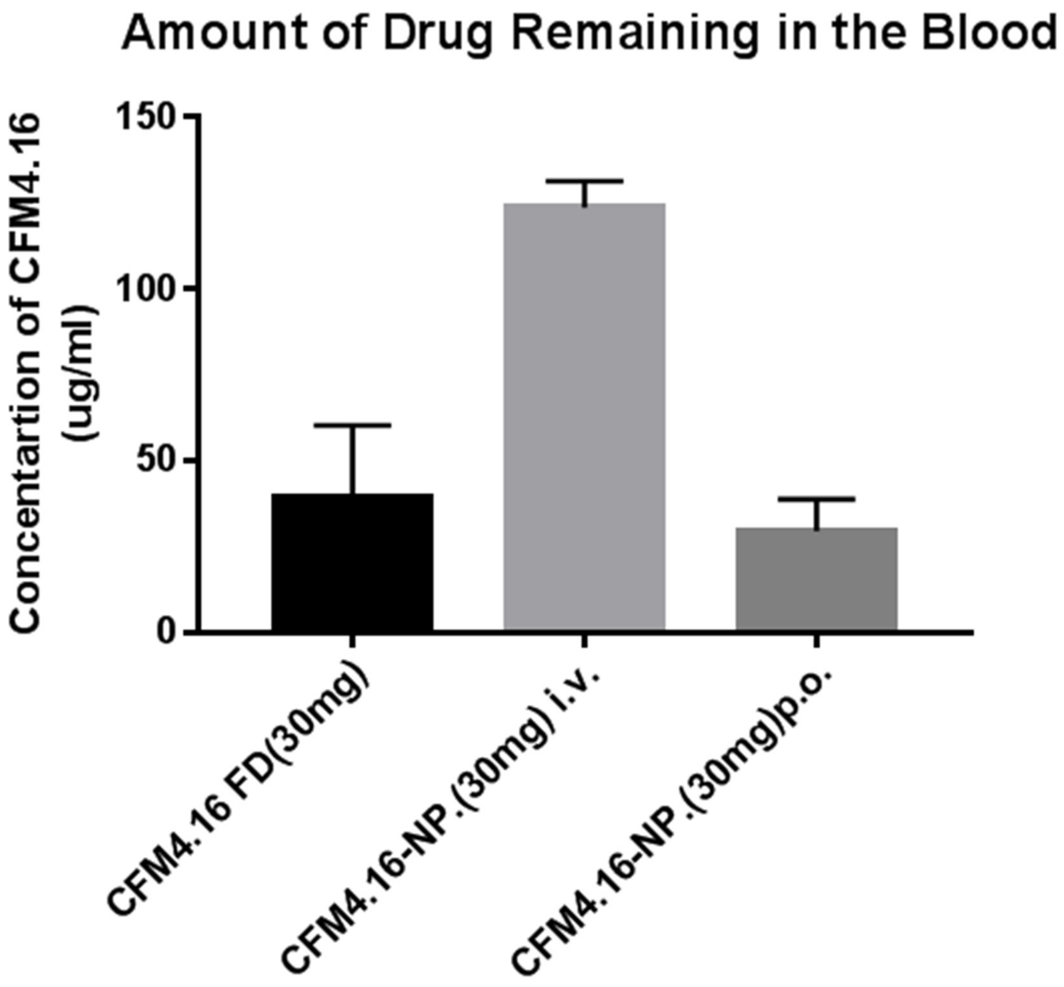

Supplementary Figure 6: Amount of CFM4.16 remaining in the blood after the last injection of drug. The histogram columns represent means of CFM-4.16 concentrations from three animals from the indicated treatment group; bars, S.E.

CFM	R^1	R^2	R^3	R^4
4 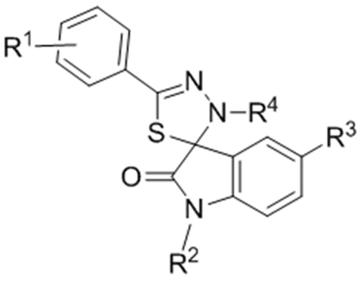	H	2-Cl-Ph-CH_2	H	H
4.16	3-Cl	2-Cl-Ph-CH_2	H	H
4.17	3-OCH_3	2-Cl-Ph-CH_2	H	H

Supplementary Figure 7: Chemical structures of CFM compounds
